# Why and How. The Future of the Central Questions of Consciousness

**DOI:** 10.3389/fpsyg.2017.01797

**Published:** 2017-10-11

**Authors:** Marek Havlík, Eva Kozáková, Jiří Horáček

**Affiliations:** ^1^National Institute of Mental Health, Klecany, Czechia; ^2^Third Faculty of Medicine, Charles University, Prague, Czechia

**Keywords:** consciousness, predictive coding, mind-wandering, default mode network (DMN), neural correlates of consciousness (NCCs)

## Abstract

In this review, we deal with two central questions of consciousness *how* and *why*, and we outline their possible future development. The question *how* refers to the empirical endeavor to reveal the neural correlates and mechanisms that form consciousness. On the other hand, the question *why* generally refers to the “hard problem” of consciousness, which claims that empirical science will always fail to provide a satisfactory answer to the question why is there conscious experience at all. Unfortunately, the hard problem of consciousness will probably never completely disappear because it will always have its most committed supporters. However, there is a good chance that its weight and importance will be highly reduced by empirically tackling consciousness in the near future. We expect that future empirical endeavor of consciousness will be based on a unifying brain theory and will answer the question as to what is the function of conscious experience, which will in turn replace the implications of the hard problem. The candidate of such a unifying brain theory is predictive coding, which will have to explain both perceptual consciousness and conscious mind-wandering in order to become the truly unifying theory of brain functioning.

## Central Questions of Consciousness

Most articles and books dealing with consciousness begin with a variation of the same sentence, which roughly claims that *conscious experience is the most intimate part of ourselves and also, probably, the most elusive problem of empirical science* (e.g., Chalmers, 1996, 1999). Over time, variations of this statement and emphasis on *elusiveness* have almost become the universal definition of consciousness in relation to subsequent methodological and conceptual skepticism (e.g., Levine, 1983; Chalmers, 1996). Even though there are many questions regarding the nature of consciousness, only the questions *why* and *how* are considered the central questions of consciousness.

The question *why is there consciousness* is closely connected to the philosophy of mind, or more precisely, to the ideas of non-reductive or even dualistic philosophers of mind. Through several epistemic arguments such philosophers introduced major epistemic gap between physical and phenomenal properties, which, according to Chalmers, represents “the central mystery of consciousness” (Chalmers, 2003, p. 104). This epistemic gap, which aims at the nature of conscious experience, has been profoundly discussed by philosophers since Nagel () used the metaphor of a bat’s mind in his essential article defending the irreducibility of conscious experience. Reduction is one of the fundamental principles of empirical science and since conscious experience defies it, the idea of the *explanatory gap* (Levine, 1983) between consciousness and empirical science began to sprout. The idea of the explanatory gap deepened with the help of several thought experiments such as the famous *knowledge argument* (Jackson, 1982), *inverted spectrum scenarios* (Shoemaker, 1982; Block, 1990) and *philosophical zombies* (Chalmers, 1996), and it finally resulted in the formulation of a distinction between *easy problems of consciousness* and the *hard problem* of consciousness (Chalmers, 1996).

The easy problems of consciousness are those that seem directly susceptible to the standard methods of cognitive science, whereby a phenomenon is explained in terms of computational or neural mechanisms. On the other hand, the hard problem refers to the nature, mechanism and role of subjective experience such as quality of deep blue (Chalmers, 1996). Hence, the *hard problem* of consciousness is the equivalent of the philosophically oriented question *why*. *Why* is a neural activity of the brain accompanied by a conscious experience despite the fact that philosophical zombies (beings with adaptive behavior without consciousness) are logically possible? Empirical science cannot hope to give a satisfactory answer to this question because it only operates within the scope of neural mechanisms (*easy problems*), which are incapable of providing a satisfactory answer to the question why is my brain activity accompanied by subjective experience (Chalmers, 1996).

Even though philosophical work in this area is interesting and quite popular, it can lead someone to very dangerous conclusion that empirical explanation of consciousness is utterly impossible. Against this conclusion stand out physicalist or even eliminative philosophers, such as (Churchland, 1989; Dennett, 1993, 2003, 2005; Bickle, 2003) and many others. For such oriented philosophers there is no such a thing as epistemic gap between physical and phenomenal properties and everything about consciousness can and will be explained by empirical science in the future. Some thinkers even attempted to *safe* consciousness as a topic from the philosophical restrictions of dualism (Brook, 2005) and reoriented themselves to the question *how.*

*How is consciousness realized by neural mechanisms* or *how do we become conscious* (Prinz, 2005, 2012) became the central question for the empirically oriented science of consciousness, whose main goal is to identify correlates of consciousness (Chalmers, 2000; Metzinger, 2000), which are the first step to describing the neural mechanisms directly responsible for the emergence of conscious experience.

The history of the empirical science of consciousness is full of proposed mechanisms. To name a few, Crick and Koch (1990) believed that the synchrony of gamma oscillations in the cerebral cortex is the key to binding and conscious experience. Edelman (1990, 2005) proposed thalamo-cortical reentrant loops as mechanisms of consciousness. Bogen (1995) argued for to the activity of the intralaminal nuclei of each thalamus. According to Llinas et al. (1994) consciousness emerges due to synchronization of thalamocortical activity below 40 Hz. Newman and Baars (1993) promoted thalamus and its reticular nucleus as the central hub for consciousness and conscious attention. His early ideas were also further developed by Dehaene and Naccache (2001), who supports the global neuronal workspace model, which claims that consciousness is a result of activity of the primary sensory cortex, and frontal and parietal areas. Prinz (2005, 2012) recently proposed his AIR theory of consciousness, which is based on the idea that consciousness emerges when information becomes available to working memory through attention, which is associated with gamma synchrony.

From the above it seems that *thalamus* and *gamma synchrony* have been considered as the primary candidates for the correlates of consciousness. The thalamus as the hot spot location for consciousness and gamma as the neural mechanism that renders neural representations conscious. Both concepts seem to be plausible. The thalamus is crucial in transmitting information from the external environment to the cortex, which itself receives relatively little other input (Saalmann and Kastner, 2011). Gamma synchrony accompanies stimulus selection under a binocular rivalry paradigm (Engel et al., 1999) and was reported during conscious detection in masking experiments (Summerfield et al., 2002; Gaillard et al., 2009). Moreover, synchronous gamma oscillations were also reported to accompany conscious auditory perception (Steinmann et al., 2014) and some even propose that gamma stands behind olfactory consciousness (Mori et al., 2013). Large-scale gamma-band synchrony was also proposed as the critical process behind conscious perception by Doesburg et al. (2009), Melloni and Singer (2010), Prinz (2012), and others. Another proposed candidate relating to the global neuronal workspace theory is P300. P300 can be elicited in tasks when subjects attend and discriminate stimuli that differ from one another in some dimension. Such discrimination produces a large positive waveform with a modal latency of about 300 ms (Sutton et al., 1965) reflecting cortical post-synaptic neuroelectric activity related to cognitive processes such as attention, stimulus evaluation and categorization. In global neuronal workspace theory, P300 is considered as a correlate of conscious access (Babiloni et al., 2006; Del Cul et al., 2007; Lamy et al., 2009; Dehaene and Changeux, 2011; Salti et al., 2012; King et al., 2014).

However, all candidates, such as the thalamus, gamma synchrony and P300, have been severely criticized lately (Aru and Bachmann, 2015; Koch et al., 2016). Conscious experience can occur without the *thalamus* in the form of hypnagogic hallucinations or hypnagogic experiences, which are present while falling asleep. During this phase, thalamic deactivation precedes the cortex, which is still immersed in complex cortical activity having direct relevance to conscious experience (hypnagogic experiences) (Magnin et al., 2010). Gamma band synchronization can occur during the phases of suppressed conscious perception (Vidal et al., 2014). P300 and gamma synchrony are not reported during task irrelevant trials, but are present and robust for task relevant stimuli, which apparently means that these candidates are responsible for post-perceptual processes and not for visual consciousness (Pitts et al., 2014a).

This gradual development is central to the question *how.* Most conclusions about consciousness are based on paradigms that are mostly directed toward the visual consciousness using experimental paradigms such as *binocular rivalry* and *visual masking.* Binocular rivalry refers to the phenomenon of ongoing competition for dominance between two different visual patterns, which are perceived by each eye separately. Both are represented in the brain, but only one is exclusively dominant for a few seconds and thus thrown into the stream of consciousness (Klink et al., 2013). Visual masking is another tool often used by conscious studies. Masking refers to a specially created condition under which a target stimulus is perceived as impaired due to another (masking) stimulus, which is presented in temporal and spatial proximity. Without the masking stimulus, the target stimulus would be perceived clearly and consciously (Ansorge et al., 2007; Bachmann and Francis, 2013). The major limitation of both approaches lies in the fact that it is not clear to what extent their results can be generalized to the entire field of conscious experience.

Moreover, the science of consciousness is now experiencing the emergence of new topics, which aim to isolate genuine correlates of consciousness from the related unconscious or other related brain processing. These new topics include *neural prerequisites* and *consequences of consciousness* (Aru et al., 2012), as well as the *reporting of conscious perception* and its *isolation from the actual correlates of consciousness* (Pitts et al., 2014a,b). Neural prerequisites and consequences of consciousness emerged from the critical idea that classical paradigms for studying consciousness relaying on contrast between conscious and unconscious perception (e.g., binocular rivalry) do not reveal genuine correlates of consciousness, since they do not include the possibilities of unconscious prerequisites and consequences. Classical paradigms, therefore, cannot from a methodological point of view never reveal the correlates of consciousness because they confound the NCC with other processes (Aru et al., 2012; Aru and Bachmann, 2015). Another topic that aims to clarify the possible confounding of NCC, is the topic of reporting. Subjective experience is private by definition, and therefore conscious perception requires a report, such as a button-press. Unfortunately, neural processing of such reports can also be confounded with genuine correlates of consciousness (Pitts et al., 2014a,b).

The science of consciousness is now experiencing a proliferation of topics that will hopefully remediate the topic of conscious experience and support the materialistically oriented understanding of conscious experience without epistemic gaps. However, another new topic that cannot be neglected is conscious *spontaneous cognition* or so-called *mind-wandering*.

## Mind-Wandering

Mind-wandering refers to the internally directed cognition, which is stimulus independent, task-unrelated, emerges spontaneously and represents significant part of our daily lives (Killingsworth and Gilbert, 2010). Interesting description, which defines mind-wandering more closely next to other types of internally directed cognition was presented in Christoff et al. (2016):

“…mind-wandering can be defined as a special case of spontaneous thought that tends to be more-deliberately constrained than dreaming, but less-deliberately constrained than creative thinking and goal-directed thought. In addition, mind-wandering can be clearly distinguished from rumination and other types of thought that are marked by a high degree of automatic constraints, such as obsessive thought” (Christoff et al., 2016).

The scientific interest in this type of internally oriented mental states is not completely new (e.g., Singer, 1966; Antrobus, 1968; Antrobus et al., 1970; Giambra, 1989). However, topic of mind-wandering experienced a new wave of interest due to the fact that this type of cognition is accompanied by the intrinsic activity of now well-known *default mode network* (DMN).

At the end of the 20th century, a series of unexpected discoveries of intrinsic brain activity was made. The authors of these discoveries were Biswal et al. (1995) and Shulman et al. (1997). Several years later, intrinsic brain activity was attributed to the influential concept of the DMN (Greicius et al., 2003; Raichle and Snyder, 2007). The DMN is a large-scale brain network coupling the medial *prefrontal cortex*; *ventral medial prefrontal cortex*; *medial and lateral temporal cortex*; *posterior inferior parietal lobule* and *precuneus/posterior cingulate cortex*. This network not only caused an essential scientific revolution in fMRI (Havlík, 2017) but also raised a new topic for studies on the conscious mental contents associated with DMN activity. To name a few, *self-referential thinking* (Gusnard and Raichle, 2001), *imagining future* (Buckner et al., 2008), *remembering and imagining future* (Addis et al., 2007), *social cognition* (Mars et al., 2012), *theory of mind* (Buckner et al., 2008; Nekovarova et al., 2014), *spontaneous cognition* (Andrews-Hanna et al., 2010), *mental time travel* (Østby et al., 2012), *internal train of thought* (Smallwood et al., 2012) and *mind-wandering* (Mason et al., 2007), which is now considered as the umbrella term for the all the above (Callard et al., 2013).

This new ‘era of wandering minds’ (Callard et al., 2013) has not yet broken into the science of consciousness. However, in the future it will be necessary to unify mind-wandering with consciousness in order to satisfactorily answer the question *how*. According to some, we spend about 20–50% of our time in mind-wandering experiences (Killingsworth and Gilbert, 2010; Song and Wang, 2012), which is a substantial part of our subjective experience that cannot be neglected. Mind-wandering represents the other side of the coin of the conscious experience next to perceptual (mainly visual) consciousness, which was the most studied topic in the science of consciousness. Identifying the mechanisms that accompany mind-wandering and render spontaneous mental states conscious will be necessary for any new universal theory of consciousness that would explain both *perceptual consciousness* and *conscious mind*-*wandering*.

One of the first methodological steps toward unifying consciousness with mind-wandering would be to examine whether candidates for conscious experience such as the thalamus, gamma band and P300 are also detectable under mind-wandering conditions. Interestingly, the thalamus, the best known cerebral region of perceptual consciousness, is not part of the DMN (from a functional connectivity point of view) and is not reported as being active during states of mind-wandering. A celebrity of intrinsic brain activity and the DMN is the *precuneus/PCC*, which is considered to be the central region of the DMN (Fransson and Marrelec, 2008; Utevsky et al., 2014). The precuneus/PCC, a major connectivity hub of brain organization, is responsible for a high degree of information integration due to its great number of connections with other regions, namely association areas and paralimbic areas (Leech and Sharp, 2014). Precuneus/PCC were proposed to be the main candidates for conscious cognitive information processing (Vogt and Laureys, 2005) and it was more recently suggested that precuneus/PCC could be very important for the balancing of internal and external attentional focus (Leech and Sharp, 2014). This could be interesting for the proponents of the idea that *attention is necessary and sufficient* for consciousness. Precuneus/PCC are also close to the ‘posterior cortical hot zone’ (Koch et al., 2016), which was recently proposed as the correlate of conscious experience.

It may be tempting to suggest that precuneus/PCC is a ‘new candidate’ for consciousness. However, that alone could be misleading and would certainly not help extinguish the *elusive nature* of consciousness. To clear up this elusiveness it would be necessary to gradually categorize the neural correlates (and later mechanisms) of both sides of conscious experience – *perceptual consciousness* and *conscious mind*-*wandering*. Given the dichotomy between externally oriented perceptual consciousness and conscious mind-wandering it is necessary to mention that both these types of our conscious life consist of the same forms of mental states such as thoughts, emotions, believes, sensory sensations, and body feelings. The only (but principal) difference is that perceptual consciousness results from the instant processing of external stimuli but mind-wandering is formed by internal manipulations of previous experiences and different levels of their neural representations. This dichotomy is also supported by the antagonistic nature of the DMN activity toward the activity of *central executive network* (CEN), which represents attention systems toward external environment (Gusnard and Raichle, 2001; Raichle et al., 2001; Raichle and Snyder, 2007) and thus relates to the perception. If DMN is active then CEN is partially disengaged and vice versa. This antagonistic nature between DMN and CEN occurs under natural conditions, however, these networks can be coupled under demanding conditions, such as goal-directed cognition (Spreng et al., 2010).

Also, determining the mechanisms of ‘attention switching’ between perceptual consciousness and mind-wandering would be very helpful for clarifying this elusiveness and the question *how*. The recently described *salience network* (SN) is a hot candidate for this mechanism. SN consists of the *anterior insula* and the *anterior cingulate cortex* and its main functions are the ‘detection of salience stimuli’ and ‘dynamical switching’ between the DMN and the *central executive network* (Menon and Uddin, 2010).

*Conscious mind-wandering* is an important part of conscious experience, and finding its mechanisms will be important for the universal theory of consciousness, which will provide a satisfactory answer to the central question *how*.

Even though an answer to the question *how* would be a great scientific discovery, it alone would not be the complete package. To get a ‘complete package of consciousness’ the second central question of consciousness *why* should not be neglected.

## Future of the Question *Why*

The future development of the question *how* is clear and the question itself does not need any serious revision. However, this does not count for the question *why.* Unfortunately, we believe that there is little hope that the most committed proponents of hard problem would be completely satisfied with conclusions of empirical science, since the core argument of hard problem is aimed at the endeavors of empirical science in the first place:

“If we show how a neural or computational mechanism does the job, we have explained learning. We can say the same for other cognitive phenomena, such as perception, memory, and language. (…) When it comes to conscious experience, this sort of explanation fails” (Chalmers, 1999, p. 12).

Proponents of the hard problem are caught in their own intuitions, which are “invulnerable (as) bedrock, an insight so obvious and undeniable that no argument could make it tremble, let alone shatter it” (Dennett, 2013, p. 312).

Even though, the hard problem is very seductive for some philosophers we believe that its strength will be seriously reduced in the near future. We have already mentioned that the question *how* needs no revision, but this does not apply to the question *why.* The first step toward this revision is to reject the ‘logical possibility of zombies’ when asking the question *why is there conscious experience.* The second step is to redirect the question *why is there conscious experience* toward the *function of conscious experience*, which it plays in the overall neural machinery. Speaking of function it should be immediately clarified what we mean. Function of consciousness should be understood clearly in the sense of physicalism or even eliminativism (as Type-A Materialism, see Chalmers, 2003) without any teleological implication. Such account of function of conscious experience should be put among the other neural functions without any privilege or special importance and thus close or even eliminate supposed epistemic gap. Unfortunately, there is no clear understanding or general consensus among philosophers and neuroscientists about the function of consciousness. This is one of the main reasons why consciousness still represents such an *elusive problem*, which has its roots in the fact that there has never been an articulated function of consciousness *based on* and *supported by* the *unifying brain theory*.

Such a unifying theory is emerging in the form of the *predictive coding framework* (PC) (Rao and Ballard, 1999) and is gaining more and more support (Hohwy, 2012, 2013; Clark, 2013, 2016; Hobson and Friston, 2014). This line of thought, which can be traced back to Immanuel Kant (Kant, 1996; Swanson, 2016), is based on the ideas of *Hermann von Helmholtz* that the brain is mainly a predictive inference machine (Von Helmholtz, 1866/1925; Dayan et al., 1995). Over time, this predictive framework has been given several names, such as *Bayesian brain* (Knill and Pouget, 2004), the *free-energy principle* (Friston, 2009) and *prediction error minimization* (Hohwy, 2013). PC claims that perception is not ‘directly’ related to the external environment, but it is created endogenously (Gregory, 1968) implicating that brain does not have to exhaustively reconstruct an environment solely by a bottom–up analysis of sensory inputs (Panichello et al., 2012). Top–down predictions, known as priors or prior knowledge, are tested against sensory inputs, which are considered as sensory evidence in the framework of PC. The differences between top–down predictions (priors) and bottom–up sensory evidence are called prediction errors. If a prediction error is encountered, the prior has to be updated according to the sensory evidence, which results in posterior beliefs. The brain of a living organism creates and uses these internal models to predict sensory inputs in order to minimize its free-energy (entropy), which is essentially a measure of surprise about sensory inputs (Friston, 2010; Friston et al., 2012; Clark, 2016).

The PC account of brain function explains perception, attention, action and other brain functions (Hohwy, 2013), and also stands as the unifying theory for other global theories of the brain (Friston, 2010). Nevertheless, for PC to be a complete and truly unifying theory of the brain, it will have to include both parts of conscious experience – *perceptual consciousness* and *conscious mind-wandering*, and explain their *function* in the terms of *prediction error minimization*. Only this will provide a satisfactory answer to the revised question *why.* A similar vision is shared by Clark (2016):

“Using this integrated apparatus might we, inch-by-inch and phenomenon-by-phenomenon, begin to solve the so-called, hard problem’ of conscious experience (…).”

Authors now widely accept that inferences and prior knowledge are crucial to understanding consciousness. It is believed that priors shape and determine the contents of consciousness and can even accelerate their access to the stream of consciousness (Melloni et al., 2011). These claims are not exaggerated. Evidence is being gathered suggesting that PC has consequences for conscious experience, such as ambiguous visual inputs (Panichello et al., 2012), predictions accelerate time of conscious access (Melloni et al., 2011; Chang et al., 2015). In addition, the well-known visual paradigms, such as continuous flash suppression (Hohwy, 2013) and binocular rivalry (Hohwy et al., 2008; Denison et al., 2011; Clark, 2013, 2016) are now being reconsidered from the methodological and explanatory perspective of PC.

However, these authors have so far been very reticent about how exactly consciousness fits into the PC framework. For now, there is an emerging idea that understands consciousness as the *final result* of unconsciousness processing. According to Hohwy (2013), *perceptual consciousness* fits in the PC theory as the ‘upshot’ or ‘conclusion’ of unconscious perceptual inferences. Melloni (2014) also expects that inferential processes are conducted behind the curtain of consciousness and what we experience are the ‘outcomes’ or ‘results’ of an unconscious inferential process. Lamme (2015) agrees with the idea that consciousness is based on the relation or more precisely conjunction of current inputs and priors, which together produce posterior beliefs. Karl Friston agrees to a certain extent with this interpretation of consciousness. According to Hobson and Friston (2016), consciousness is not a phenomenon but a process, a process of optimizing beliefs through inference (consciousness, dreams, and inference; the Cartesian theater revisited; a response to our theater critics).

Consciousness may emerge from the interaction of unconscious streams – top–down predictive processing and unconscious bottom–up sensory processing. But what is the nature of the contents of conscious experience?

There are two possible but strictly opposing concepts of the relation between predictive error and consciousness. At first glance, the contents of subjective experience could be understood as being composed solely of prediction errors, which are the most important for the updating of priors (adaptive learning). What is repeatedly correctly predicted is fully adaptive and thus does not need to be perceived or be part of conscious experience. This would, however, mean that conscious agents subjectively operate in constant surprise and constant entropy. It is, therefore, more likely that *contents* of *conscious perception* are formed by representations that best predict the environment and sensory inputs. Correctly predicted/inferred states are fully adaptive, such contents are still part of the conscious experience, but there is no need for them to be specially addressed within conscious experience. Correctly predicted states are not needed for the actualization of priors and, therefore, are evaluated as dull and not salient. What stands out in the stream of conscious experience as the most salient states are prediction errors. Conscious experience is not composed purely of prediction errors, but prediction errors that are not unconsciously inferred are the most salient inputs, which get the privilege in the stream of conscious experience. Prediction errors that stand out from other inferred states are priors about to be updated. This could be the future answer to the question of the function of conscious experience.

Even though *conscious perception* has already its place in PC theory, *conscious mind-wandering* is still mostly left out of this discussion. On the one hand, this is understandable since the resurrection of this theme emerged only few years ago thanks to the DMN, but, on the other hand, the future synthesis of these themes will be necessary. If PC will not include conscious mind-wandering into its explanation of brain behavior, it will not only fail in the explanation of consciousness, it will also fail as the global unifying brain theory.

In PC theory, every agent’s mental state is considered as a result of self-preservation processes and such a theory cannot neglect inner conscious states, which represent large part of an agent’s mental life. Recently, PC was put into context with sleep and dreaming consciousness, whereby its function is the optimization of the internal model of the world by removing its redundant parameters and thus minimizing its complexity (Hobson and Friston, 2012, 2014). PC framework has been also applied to interoception, i.e., “sense of internal physiological condition of the body” (Seth, 2014, p. 1). In this view, emotional feelings can be understood as interoceptive inferences. “Emotional content is determined by a suite of hierarchically organized generative models that predict interoceptive responses to both external stimuli and the internal signals controlling bodily physiology” (Seth and Critchley, 2013, p. 48). Such combination of top–down inferences on the external (exteroception) and internal (interoception) causes of changes in bodily signals constitute the base of experience of selfhood (Seth, 2013). These authors propose a mechanistic model of subjective sense of presence – a basic property of normal conscious experience – which is related to the activity of anterior insular cortex (Seth et al., 2012).

Another recent hypothesis says that in order to reduce free-energy, the brain uses spontaneous activity to obtain maximum information transfer and at the same time to minimize metabolic costs as much as possible (Tozzi et al., 2016). The basis of PC is that the brain is directed to the construction of its own prior expectations of the world in order to reduce its free-energy. Apparently, the same goes for the neural systems that are hierarchically higher. These higher neural systems also try to suppress the free energy of hierarchically lower neural systems. DMN is supposed to occupy the highest point in this neural hierarchy and, therefore, provides top–down control of so-called attentional networks (in gamma power) and minimizes their free-energy (Carhart-Harris and Friston, 2010).

There are two ways that agents can suppress free energy (surprise). The first is by *changing sensory input* by acting on external states and the second is by *modifying their internal states* (Friston, 2010; Tozzi et al., 2016). DMN, consisting of memory regions (Buckner et al., 2008) and mainly active during resting states, is known to be associated with many mind-wandering mental states and mental time travel (e.g., Andrews-Hanna et al., 2010; Østby et al., 2012; Smallwood et al., 2012). The function of conscious mind-wandering could be the active, conscious modifying and optimization of internal models of the world, not by reducing their complexity (Hobson and Friston, 2012), but by creating plausible priors/predictions (based on memories) to reduce possible free energy in the future. In this respect, mind-wandering would have the opposite role to sleep and dream consciousness, which are thought to minimize the complexity of inner models (Friston, 2012; Hobson and Friston, 2014).

Daniel Dennett on one occasion spoke about so-called *Popperian creatures*, who possess an *inner environment* that permits them to sacrifice their hypotheses instead of them and on this basis permits them to take the best possible future actions (Dennett, 1997). This seems close to the idea of the brain as a predictive machine which can “generate virtual realities (hypotheses) to test against sensory evidence” (Hobson and Friston, 2012). The function of conscious mind-wandering may be a by-product of a brain that does not want to wait for a future surprise and, therefore, tries to minimize it during the resting states (period of training) that accompany endogenous activity.

Karl Friston said, “the future of imaging neuroscience is to refine models of the brain to minimize free-energy, where the brain refines models of the world to minimize free-energy” (Friston, 2012, p. 1230). If free-energy minimization is the brain’s main function, how else should the brain spend its resting states than in states of active inference and testing its hypotheses/models against possible scenarios (future external states) and thus optimize its priors. The brain in PC is understood as a good model of its environment, but this model should not be strict but flexible, so the brain should actively infer many possibilities that could occur in its environment. Rehearsing the learned facts about the world and testing new hypotheses against this in safe *conscious mind-wandering states* sounds quite possible. Therefore, the possible function of the *conscious mind-wandering* is *conscious optimization* and *maximization* of the complexity of priors.

## Conclusion and Future Directions

Above, we have drafted the possible development of the science of consciousness. First, we propose that the initial step should be to replace the methodologically hardly approachable research on the explanatory gap between consciousness and empirical neuroscience by the question on the adaptive role of consciousness (“why”). Second, we argue that empirically oriented studies of consciousness should address not only perceptual consciousness but also *mind-wandering representing* the other side of conscious experience. Consequently, any current or future candidates for the neuronal correlates of consciousness should be plausible and testable for both modes of conscious experience. Third, *prediction error minimization* represents the hot candidate for both unifying theory of brain function and development of conscious experience. However, future empirical research in this field should primarily answer the open question if content of consciousness represents the best predicts (priors) of the environment/inputs, or if it corresponds with constant surprise (set of prediction errors). This answer will elucidate the adaptive function of consciousness (why) and underlying neuronal mechanism (how), only if it is valid for both perceptual consciousness and mind-wandering. We assume that empirical approaches to these questions might substantially contribute to our understanding the nature of consciousness. Hopefully, we will then be able to refrain from beginning our articles by mentioning the elusive nature of consciousness and this elusiveness will be replaced by a sentence about the old philosophical relic of consciousness, the hard-problem.

## Author Contributions

MH wrote the article. EK and JH revised it and contributed with critical comments and suggestions. All authors have approved the final content of the manuscript.

## Conflict of Interest Statement

The authors declare that the research was conducted in the absence of any commercial or financial relationships that could be construed as a potential conflict of interest.
